# Impaired Fibrinolysis in Angiographically Documented Coronary Artery Disease

**DOI:** 10.1155/2015/214680

**Published:** 2015-02-23

**Authors:** Adriano Basques Fernandes, Luciana Moreira Lima, Marinez Oliveira Sousa, Vicente de Paulo Coelho Toledo, Rashid Saeed Kazmi, Bashir Abdulgader Lwaleed, Maria das Graças Carvalho

**Affiliations:** ^1^Faculty of Pharmacy, Federal University of Minas Gerais, Avenida Antonio Carlos 6627, 31270-901 Belo Horizonte, MG, Brazil; ^2^Department of Haematology, University Hospital Southampton, Southampton, UK; ^3^Faculty of Health Sciences, University of Southampton, Southampton, UK

## Abstract

Impaired fibrinolysis may predispose to coronary artery disease (CAD). Hypofibrinolysis due to high levels of plasminogen activator inhibitor-1 (PAI-1) has been reported in CAD. A novel regulator of fibrinolytic activity, thrombin activatable fibrinolysis inhibitor (TAFI), has attracted attention in recent years. It acts by blocking the formation of a ternary complex of plasminogen, fibrin, and tissue plasminogen activator (t-PA). Previously ambiguous results regarding TAFI levels have been reported in CAD. We measured plasma levels of PAI-1 and TAFI antigen in 123 patients with age ranging from 40 to 65 years who had been submitted to coronary angiography and assessed the association of these markers with the extent of stenosis in three groups: angiographically normal artery (NAn), mild to moderate atheromatosis (MA), and severe atheromatosis (SA). Plasma levels of PAI-1 were increased in patients with severe atheromatosis compared to mild/moderate atheromatosis or to normal patients (66.60, 40.50, and 34.90 ng/mL, resp.; *P* < 0.001). For TAFI no difference was found between different groups. When patients were grouped in only two groups based on clinical cut-off point for intervention (stenosis less than or above 70%) we found increased plasma levels for PAI-1 (37.55 and 66.60 ng/mL, resp.; *P* < 0.001) and decreased plasma levels for TAFI (5.20 and 4.53 *μ*g/mL, resp.; *P* = 0.04) in patients with stenosis above 70%. No difference was found in PAI-1 or TAFI levels comparing the number of affected vessels. *Conclusion*. As evidenced by a raised level of PAI-1 antigen, one can suggest an impaired fibrinolysis in stable CAD, although no correlation with the number of affected vessels was found. Curiously, a decreased plasma level of total TAFI levels was observed in patients with stenosis above 70%. Further studies measuring functional TAFI are required in order to elucidate its association with the extent of degree of atheromatosis.

## 1. Introduction

The endothelium mediates a variety of vital physiological functions. While in health it maintains vascular integrity by expressing vasoprotective and thromboresistant molecules, on activation endothelial cells (ECs) acquire a phenotype that promotes atherosclerosis [1]. The thrombin catalyzed conversion of plasma fibrinogen into fibrin is the final step of coagulation cascade during haemostasis. The formation of thrombus is followed by the process of fibrinolysis which consists of an enzymatic dissolution of the fibrin clot by plasmin. It is controlled by endothelial cells through secretion of physiological plasminogen activators like tissue type plasminogen activator (t-PA) and urokinase type plasminogen activator (u-PA). Fibrinolysis is initiated when both plasminogen and t-PA bind to fibrin surface to generate plasmin [2]. However, plasmin is generated on the surface of endothelial cells in the presence or absence of fibrin. In the absence of fibrin plasmin generation is dependent on the constitutively expressed plasminogen and t-PA receptor, annexin 2A. The endothelium also exerts an inhibitory effect on fibrinolysis through the synthesis of an inhibitor, type 1 plasminogen activator inhibitor (PAI-1). High levels of PAI-1 have been shown to be associated with CAD [3, 4]. Yet another fibrinolytic inhibitor is thrombin activatable fibrinolysis inhibitor (TAFI), a plasma zymogen that potently inhibits fibrinolysis when converted to an active enzyme by thrombin, plasmin, trypsin, and, more efficiently, thrombin-thrombomodulin complex [5]. Activated TAFI (TAFIa) inhibits fibrinolysis by removing the carboxyterminal lysine (and arginine) residues on partially degraded fibrin, blocking the formation of a ternary complex of plasminogen, and t-PA. Some studies have shown a trend for increased TAFI levels in CAD patients [6–8]. On the contrary other investigators have found decreased levels of TAFI in CAD patients [9].

The aim of this study was to investigate the association of PAI-1 and TAFI antigen levels with increasing degrees of coronary atheromatosis in patients undergoing angiography.

## 2. Material and Methods

The population investigated consisted of 123 subjects with age ranging from 40 to 65 years, who had been consecutively submitted to coronary angiography in the Department of Haemodynamics of Socor Hospital, Belo Horizonte, Brazil. This protocol was submitted to the local ethical committees in research of Socor Hospital and Federal University of Minas Gerais. Signed informed consent was required for all subjects enrolled in this study.

This study assessed a population of intermediate to high risk of CAD who had been referred for catheterization due to worsening clinical features on a background of a history of stable angina. Patients with acute coronary syndrome in the preceding 3 months were excluded from the study as were those having concomitant treatment with anticoagulants, lipid lowering drugs, or estrogens, patients with known bleeding or thrombotic disorders, and those with renal, hepatic, autoimmune, or malignant diseases.

Coronary angiography was performed in all 123 subjects by percutaneous transfemoral approach. The images were recorded digitally and all angiograms were analyzed by three experienced cardiologists. The patients were grouped according to the angiographic findings as follows: no stenosis (Group I), stenosis of up to 30% of the luminal diameter in at least one coronary artery (Group II), stenosis of 30 to 70% of the luminal diameter in at least one coronary artery (Group III), and stenosis of more than 70% of the luminal diameter in at least one coronary artery (Group IV).

Blood samples were collected from 12 h fasting patients after coronary angiography (since patients stratification was dependent on this procedure), however, before any other intervention following angiography, into vacuum tubes containing 3.2 w/v sodium citrate as anticoagulant. Blood samples were immediately centrifuged at 2100 g for 20 minutes and plasma samples were separated and stored at −70°C until analysis.

Plasma level of TAFI was performed using a commercially available ELISA Kit (TAFI Antigen Kit, Affinity Biologicals Inc., Canada) according to the manufacturer's instructions.

Quantitative determination of plasma TAFI was performed using citrated set diagnostic Visualize TAFI Antigen Kit (Affinity Biologicals Inc., Canada) whose analytical principle is the enzyme-linked immunosorbent assay (ELISA capture), strictly following the instructions provided by the manufacturer. A microplate reader, BIO-RAD 550-USA, was used for reading the reaction. The reference curve was performed using the standard provided by the manufacturer obtaining the points of 6.8, 3.4, 1.7, 0.850, 0.425, and 0.213 mg/mL and two plasma controls provided by the kit were used to verify the assay performance. The concentration of TAFI in the sample was obtained using the following equation:

(1) $$\begin{array}{c} \log\left(y\right)=A+Bx\log\left(x\right), \end{array}$$

with reference value from 5.8 to 10.0 *μ*g/mL.

PAI-1 level was determined using a commercial ELISA Kit (IMUBIND PAI-1 ELISA Kit, American Diagnostica Inc., USA). The intra- and interassay coefficients were 9.0% and 6.6%, respectively (IMUBIND PAI-1 ELISA Kit, American Diagnostica Inc., USA). For both, TAFI and PAI-1, control plasmas were used to verify the assay performance.

Normal distribution of the data was checked by Shapiro-Wilks test. The results were presented as mean and standard deviation (SD) when normally distributed and otherwise as median and interquartile ranges (25th and 75th percentiles). For normally distributed data, ANOVA was used to compare three groups. Mann-Whitney and Kruskal-Wallis tests followed by Dunn's test were used in case of non-normally data for comparison of three groups. Statistical analysis was performed by Sigma Stat version 1.0 software system. A value of *P* < 0.05 was chosen for statistical significance.

## 3. Results

The baseline characteristics of the study participants are presented in Table 1. The three groups showed homogeneity in relation to age, sex, and body mass index and no statistically significant difference was noted. The incidence for hypertension was high in all three groups. The differences in the incidence of smoking, sedentary lifestyle, and previous history of CAD were statistically significant amongst different degrees of severity of CAD.

Plasma levels of PAI-1 were increased in patients with severe atheromatosis (Group IV) compared to mild/moderate atheromatosis (Groups II/III) (*P* < 0.001) or to angiographically normal (Group I) patients. For TAFI no statistically significant difference was found between groups. Plasma levels of PAI-1 and TAFI are presented in Table 2.

Considering that a clinical intervention must be made in all patients presenting with stenosis above 70%, all of them were rescored in two groups: stenosis of up to 70% of the luminal diameter in at least one coronary artery and stenosis of more than 70% of the luminal diameter in at least one coronary artery.  Table 3 shows results for PAI-1 and TAFI in these two groups.

Based on this clinical cut point for intervention, we found* increased* plasma levels for PAI-1 (*P* < 0.001) and decreased plasma levels for TAFI (*P* = 0.04) in patients with stenosis of more than 70%.

We also explored the severity of CAD through the number of affected vessels in patients with stenosis of more than 70% versus plasma levels of PAI-1 and TAFI. No statistically significant difference was found between groups (Table 4).

## 4. Discussion

Regulation of blood coagulation is an intricate coordination between different pathways. Maintaining a balance between thrombin-stimulated fibrin clot formation and plasmin-induced clot lysis is essential for optimal haemostasis. Any disturbance in these pathways causes a haemorrhagic or a thrombotic tendency depending on the shift of the balance [10]. The data from this study demonstrate that fibrinolysis is impaired in subjects with angiographically documented CAD. PAI-1 levels were increased in patients with severe atheromatosis compared to mild/moderate atheromatosis (*P* < 0.001) or angiographically normal patients.

Increased levels of PAI-1 have been described in patients with CAD after myocardial infarction (MI) [3] and are considered a risk factor for recurrence [4]. We tried to correlate the severity of disease with the number of affected vessels and PAI-1 levels, but no significant difference between different groups was found (Table 4). Thrombin also plays an important role in fibrinolytic modulation through activation of TAFI [11]. Activated TAFI inhibits plasmin formation and downregulates fibrinolysis [5] contributing to a hypofibrinolytic state in cardiovascular disease [9]. In the present study, we did not find any significant difference in TAFI levels between groups (Table 2). Interestingly, when we rescored groups based on clinical cut point for intervention (stenosis of more than 70%) TAFI plasma levels were decreased in subjects with stenosis of more than 70% (Table 3) compared to those with stenosis of up to 70%, consistent with a previous study [9]. No difference was found between severity of disease through number of affected vessels and TAFI levels (Table 4). Controversial results regarding TAFI levels in CAD patients have been described possibly because of both different characteristics of patients investigated and methods used for its determination [12].

Different clinical studies have investigated the possible relationship between TAFI and cardiovascular events [12]. The results have been inconsistent and studies have reported high, normal, and low plasma levels of TAFI [6–8].

We could speculate that the amount of TAFI generated during coagulation and fibrinolysis varies in different cardiovascular disease stages and, in addition, different results obtained through different assays demonstrate the variable reactivity of antibodies toward different isoforms of TAFI. An important study [13] comparing different TAFI assays has demonstrated that pro-TAFI assay measures high levels of TAFI in CAD, while TAFI antigen assays can detect no alterations in TAFI plasma levels or a slight decrease of TAFI plasma levels in the same samples. We can also speculate that different antibodies react to different isoforms of TAFI, yielding ambiguous results.

The importance of haemostatic alterations in CAD is being increasingly recognised in cardiovascular diseases. Our study has demonstrated that PAI-1 plasma levels are increased in a limited number of CAD patients in Brazilian patients, in line with previous studies [5, 9]. Although decreased TAFI levels are in agreement with other studies [9], its physiological relevance and pathogenic role remain unclear since only the levels of TAFI antigen were evaluated precluding any conclusion on the TAFI function.

The major limitation of the present study was that our observations were based on a small number of patients, which precludes a specific cut-off for PAI-1 and TAFI antigens in severe disease. Another limitation is the fact that in a cohort cross-sectional study we tried to evaluate associations, not predictions or causation. Finally, our findings are related to a group of patients referred for catheterization due to thoracic pain selected by experienced cardiologists. Thus, our subjects with no stenosis may not be representative of the general population. Further work is required to investigate PAI-1 and TAFI antigens roles in CAD in a greater number of patients and functional assays are also required for clarifying the real role of these fibrinolysis inhibitors. Also known modifiers of PAI-1 levels such as smoking, body mass index, and circadian variation should be considered for result interpretation.

In conclusion, this small study confirms an impaired fibrinolysis in stable CAD considering the increased levels of PAI-I, indicating that this marker is actually associated with the atheromatosis extent, although the number of affected vessels seems not to have contributed to increase of PAI-1 levels.

## Figures and Tables

**Table 1:** Baseline patients' characteristics.

	Group I	Groups II/III	Group IV	*P*
*n* (M/F)	35 (16/19)	31 (17/14)	57 (31/26)	ns
Men	16 (45.7%)	17 (54.8%)	31 (54.4%)	ns
Age (years)	59.0 ± 7.5	59.5 ± 9.0	60.5 ± 8.8	ns
BMI (Kg/m^2^)	25.3 ± 4.1	26.8 ± 4.7	25.8 ± 3.5	ns
Current smoker	6 (17.1%)	8 (25.8%)	23 (40.4%)^a^	*P* ^a^ = 0.020
Hypertension	31 (88.6%)	25 (80.6%)	48 (84.2%)	ns
Sedentary lifestyle	33 (94.3%)	23 (74.2%)^A^	43 (75.4%)^a^	*P* ^a^ = 0.021 *P* ^A^ = 0.023
Family history of CAD	14 (40.0%)	18 (58.1%)	29 (50.8%)	ns
Diabetes mellitus	5 (14.3%)	7 (22.6%)	8 (14.0%)	ns
Previous history of CAD	7 (20.0%)	12 (38.7%)	35 (61.4%)^a^	*P* ^a^ < 0.0001

*n* = sample size; M = male; F = female; BMI = body mass index; ns = not significant; Group I = normal; Groups II/III = mild/moderate atheromatosis, up to 70% in at least one coronary artery; Group IV = severe atheromatosis, >70% in at least one coronary artery; a/A = significant difference to Group I (ANOVA).

**Table 2:** Plasma fibrinolytic markers.

	Group I	Groups II/III	Group IV	*P*
*n*	35	31	57	—
PAI-1	34.90 (27.86; 42.43)	40.50 (34.24; 51.83)	66.60^a,b^(44.70; 91.65)	*P* < 0.001
TAFI	5.71 ± 1.73	5.21 ± 1.42	4.97 ± 1.41	ns

*n* = sample size; Group I = normal; Groups II/III = mild/moderate atheromatosis, up to 70% in at least one coronary artery; Group IV = severe atheromatosis, >70% in at least one coronary artery; PAI-1 = type 1 plasminogen activator inhibitor; TAFI = thrombin activatable fibrinolysis inhibitor; ^a^versus Group I and ^b^versus Groups II/III; ns = not significant.

Values for PAI-1 are given in median (25th and 75th percentiles) and expressed in (ng/mL).

Values for TAFI are given in mean ± SD and expressed in (*μ*g/mL).

**Table 3:** Plasma fibrinolysis markers considering stenosis of 70% as clinical cut point for intervention.

	Stenosis < 70%	Stenosis > 70%	*P*
*n*	66	57	—
PAI-1	37.55 (30.90; 49.20)	66.60^a^ (44.70; 91.65)	*P* < 0.001
TAFI	5.20 (4.37; 6.31)	4.53^a^ (4.04; 5.63)	*P* = 0.04

*n* = sample size; PAI-1 = type 1 plasminogen activator inhibitor; TAFI = thrombin activatable fibrinolytic inhibitor; ^a^versus stenosis <70%. Values for PAI-1 and TAFI are given in median (25th and 75th percentiles) and expressed in ng/mL and *μ*g/mL, respectively.

**Table 4:** Plasma fibrinolysis markers considering number of affected vessels.

	1v	2v	3v	*P*
*n*	16	13	28	—
PAI-1	92.79 ± 60.45	71.83 ± 39.29	62.15 ± 31.97	ns
TAFI	5.31 ± 1.52	4.75 ± 1.16	4.87 ± 1.46	ns

*n* = sample size; 1v = stenosis in one vessel; 2v = stenosis in two vessels; 3v = stenosis in three or more vessels; PAI-1 = type 1 plasminogen activator inhibitor; TAFI = thrombin activatable fibrinolytic inhibitor; ns = not significant. Values for PAI-1 and TAFI are given in mean ± SD and expressed in ng/mL and *μ*g/mL, respectively.
